# Spatiotemporal Variability of Trace Elements Fingerprints in Otoliths of Japanese Eel (*Anguilla japonica*) and Its Use in Tracing Geographic Origin

**DOI:** 10.3390/biology11121733

**Published:** 2022-11-29

**Authors:** Takaomi Arai, Shogo Kimura

**Affiliations:** 1 Environmental and Life Sciences Programme, Faculty of Science, Universiti Brunei Darussalam, Gadong BE 1410, Brunei; 2 Graduate School of Frontier Sciences, The University of Tokyo, Kashiwa 277-8564, Japan

**Keywords:** element, environmental forensics, otolith microchemistry, stock identification, traceability

## Abstract

**Simple Summary:**

Discrimination of geographic origins in seafood is crucial to safeguard and control food security and quality for consumers. We could successfully discriminate the origins of wild and cultured freshwater eel, *Anguilla japonica*, which is one of the most valuable fish in East Asia with high market demand, by means of trace elements’ fingerprints (TEF) in the otoliths. Spatiotemporal variability of TEF was examined in the eels collected in wild and cultured environments in East Asia. Discriminant function analysis showed less temporal variation but high spatial variation, suggesting that otolith TEF reflect each habitat or aquaculture environment. The otolith TEF can be a reliable tool to discriminate the geographic origin of the fish.

**Abstract:**

To secure traceability along supply chains of foodstuffs, the spatiotemporal variability of trace elements’ fingerprints (TEF) in fish otoliths provides a powerful tool to determine and discriminate the origin. Spatiotemporal variability of TEF was examined in a commercially important seafood, Japanese eel (*Anguilla japonica*), by means of laser ablation-inductively coupled plasma mass spectrometry (LA-ICPMS). Six elemental ratios (Na:Ca, Mg:Ca, P:Ca, K:Ca, Sr:Ca, and Ba:Ca) were determined in the otoliths of specimens originating from four aquaculture farms to examine the spatial variability and from one wild habitat over three years to examine the temporal variation. Significant temporal variation was found in Mg:Ca and Sr:Ca ratios; however, discriminant function analysis showed a lower temporal variation (50%) for the three years. Spatial variations were significant in Sr:Ca and Ba:Ca ratios, and discriminant function analysis showed high (80%) spatial variation among the four farms. Otolith TEF in the Japanese eel showed specific spatial variation among aquaculture farms but intangible temporal variation, suggesting the otolith TEF reflect each aquaculture environment. The present study shows that otolith TEF can be a reliable tool to discriminate the geographic origin of the Japanese eel.

## 1. Introduction

Determination of geographic origins in foodstuffs is essential to control food quality and safeguard consumers [1,2,3]. Today, the globalization of seafood trading has accelerated the diverse movements of foodstuffs from producers to consumers [1,2,3]. The labeling of the country of origin in seafood production and processed foods is required in many countries [1,2,3]. Consumers generally refer and pay attention to the origin labeled on commercial products [1,2,3]. However, fraudulent claims of product origin and illegal commercialization and trafficking of seafood are global concerns in the world seafood trade [1,2,3]. These issues lead to a severe deficiency in transparency and traceability in the seafood trade throughout the global supply chain. Therefore, biochemical and genetic markers to identify the species and origin can be useful for ensuring the validity of seafood labeling [4,5,6,7].

Trace elements’ fingerprints (TEF) are widely used to discriminate the geographic origins of seafood [8,9,10]. Differences and variabilities of the elemental compositions that correspond to the region of origin are crucial to discriminate derivation and provenance. In the teleost fish, TEF in the otolith have been applied considerably during the past decade as otolith chemistry reflects the permanent record of the chemical and physical qualities of the ambient environment experienced by individual organisms [11,12,13]. Otoliths are calcified structures and incorporate various biotic and abiotic information perpetually during fish growth [11]. Fish otoliths can be used as a time recorder of environmental conditions over time. Otolith microchemistry has revealed life history events, migration patterns, and stock structure in many fish species up to now [8,10,11,12,13,14]. Furthermore, otolith TEF can be used to discriminate natal origin and to determine the relative contribution of different nursery areas to mixed adult stocks [15,16,17].

A freshwater eel species, the Japanese eel (*Anguilla japonica*) is a catadromous fish species which is of high value to commercial fisheries and aquaculture in East Asia [18,19]. A single spawning area for the species has been found in the western North Pacific region, and molecular genetic studies have confirmed that the Japanese eel forms a panmictic population [20]. Currently, artificial propagation of the Japanese eel has not yet been successful at the commercial level and hence, all cultured individuals rely on wild juveniles (glass eels) [21]. Therefore, all Japanese eels include wild and cultured individuals belonging to the same population, which hinders genetic discrimination at the spatiotemporal level. However, eels in wild and aquaculture environments undergo different water environments. The differences experienced in each wild habitat and aquaculture farm are recorded into the eel otoliths.

Otolith elemental compositions are affected by abiotic factors such as salinity and temperature, and biotic factors such as ontogeny and physiology, and the interactive effects are crucial to reconstruct the environment that each fish experienced [11,12,13,14,22,23,24,25]. TEF are broadly used as natural tags to discriminate the population and the provenance in teleost fish otoliths [15,16,17]. There have been many studies applied using otolith elemental composition to reveal the life history traits and migratory histories of the Japanese eel [26,27,28,29] and other eel species [30,31,32,33]. However, little application of TEF to discriminate the population and the origin has been executed in the Japanese eel and other eels.

In the present study, we examined the spatiotemporal variations in the otolith elemental composition in the Japanese eel (*Anguilla japonica*) collected in wild and aquaculture environments. We first examined the temporal variations in the elemental composition using wild eels for three years. Secondly, spatial variations were assessed in eel otoliths collected from aquaculture farms in East Asia together with a wild habitat. We also discussed the validity and usefulness of TEF in otoliths to discriminate the geographic origin of the Japanese eel.

## 2. Materials and Methods

Our protocols followed the ethical guidelines for the use of animals of Universiti Brunei Darussalam (UBD) and were approved by the Animal Ethics Committee at UBD (Approval Code: UBD/RSCH/1.4/FICBF(b)/2021/037; Approval Date: 15 September 2021).

A total of 137 otoliths in the Japanese eel were used in the present study (Table 1). To examine the temporal variability of TEF, 70 otoliths were used in a wild environment, Oi River, Shizuoka Prefecture, Japan (30 otoliths in 2007, 20 otoliths in 2008, and 20 otoliths in 2009) (34°53′28″, 138°5′42″–35°2′40″, 138°5′18″) (Table 1). Of the 70 specimens, 4 were males, and 66 of the 70 specimens were females. The spatial variability of TEF was examined using otoliths collected from four aquaculture farms from East Asia: 19 otoliths in China; 14 otoliths in Kagoshima Prefecture of Japan; 14 otoliths in Shizuoka Prefecture of Japan; and 20 otoliths in Taiwan (Republic of China) (Table 1). All cultured eels were males. We also examined the spatial variability of TEF using 70 otoliths of wild eels together with the otoliths from the four aquaculture farms. After measuring the total length (TL), sagittal otoliths were extracted from all eels, cleaned in an ultrasonic bath with Milli-Q water, rinsed in Milli-Q water, and then air-dried. Seven isotopes, ^23^Na, ^24^Mg, ^31^P, ^39^K, ^43^Ca, ^88^Sr, and ^138^Ba, were measured in the edge areas which reflected the recent environmental history in each otolith using laser ablation-inductively coupled plasma mass spectrometry (LA-ICP-MS). The 7500CS ICP-MS (Agilent Technologies, California, USA) connected with the 213 nm Nd-YAG laser ablation system (New Wave Research Inc., California, USA) was used as the LA-ICP-MS system. The He flushing technique and a stabilizer device were applied to improve the measurement accuracy [34].

Four standard materials, NIST 612 standard glass (National Institute of Standards and Technology, Maryland, USA), a clear calcite with known composition [8], and powdered coral (JCp-1) and powdered giant clam (JCt-1) distributed by the National Institute of Advanced Industrial Science and Technology (Tsukuba, Japan) [8], were used to calibrate from the signal intensity to the element. These standards were chosen following the elemental properties of each standard material in each analysis.

Duplicate 55 μm diameter laser beam spot measurements with 10 Hz in the frequency of the laser beam were conducted at the edge of each otolith. The elemental data were shown as the mean of 2 spots [8]. Standard references and background levels were determined before and after each scan. The isotope ^43^Ca was used as an internal standard, and all elemental data were presented as their molar ratio to Ca. Mean estimates of precision (%, relative standard deviation) in each standard were less than 10% for all elements.

Spatiotemporal variability of trace elements fingerprints (TEF) in eel otoliths over three years, among four aquaculture farms, and between wild (one site) and aquaculture (four sites) environments were examined by means of ANOVA, and thereafter Tukey and Kramer’s multiple comparisons among years and locations. Quadratic discriminant function analysis (QDFA) of multi-signatures for each sampling group was applied to examine whether otolith TEF discriminate their habitat (pond) differences. The capacity of otolith elemental compositions to discriminate habitat differences based on classification ratio (%) using QDFA with jackknife cross-validation was tested [8]. TLs over three years from a wild habitat and among four aquaculture farms were examined using ANOVA, and thereafter Tukey and Kramer’s multiple comparisons among years and locations. The significance of the correlation coefficient and regression slope between otolith elemental ratios and TL were tested by Fisher’s Z-transformation and by analysis of covariance (ANCOVA). Difference in otolith elemental ratios between male and female in wild eels was tested using the Mann–Whitney *U*-test.

## 3. Results

**Figure 1:**
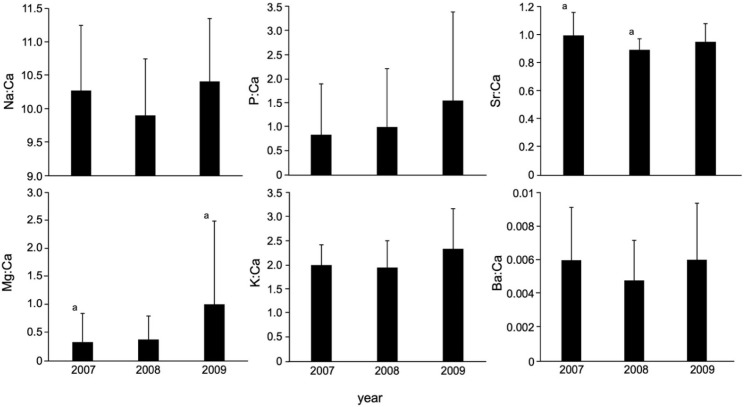
Otolith elemental ratios (mean ± SD) for three years (2007, 2008, 2009) in the Japanese eel (*Anguilla japonica*) collected in Oi River, Shizuoka Prefecture, Japan. The letter a indicates statistically significant difference at *p* < 0.05.

**Figure 2:**
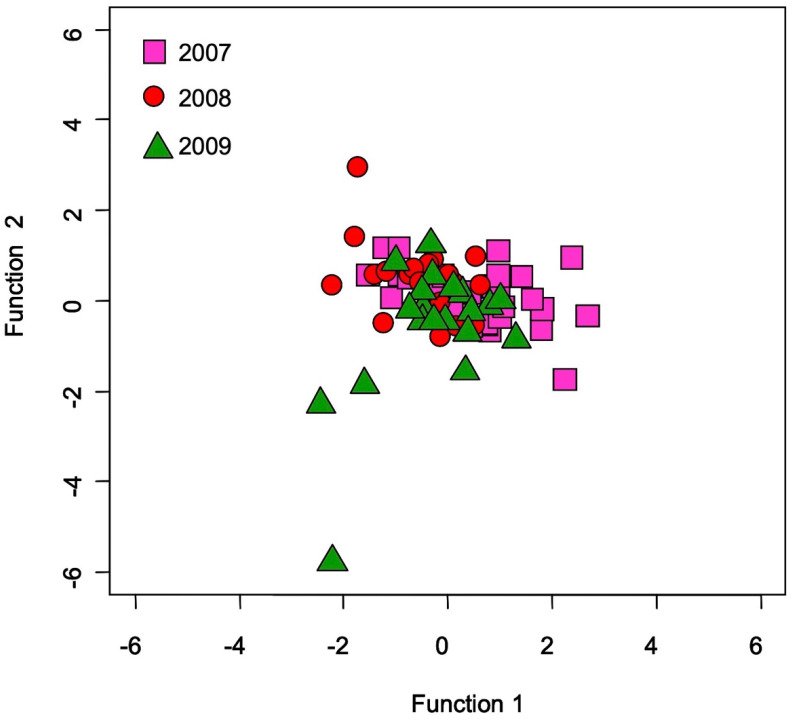
Quadratic discriminant function analysis over three years (2007, 2008, 2009) based on all the otolith elemental ratios (Na:Ca, Mg:Ca, P:Ca, K:Ca, Sr:Ca, and Ba:Ca) in the Japanese eel (*Anguilla japonica*) collected in Oi River, Shizuoka Prefecture, Japan.

In testing for temporal variability of TEF over three years, there were significant differences found in Mg:Ca and Sr:Ca (*p* < 0.05, df = 2, F = 3.555), while no significant differences were found in Na:Ca, P:Ca, K:Ca, and Ba:Ca (*p* > 0.05, df = 2, F = 1.424–2.572) (Figure 1). In Mg:Ca and Sr:Ca, significant differences were found between 2007 and 2009 (*p* < 0.05) and between 2007 and 2008 (*p* < 0.05), respectively, while no significant differences were found between other years (*p* > 0.05) (Figure 1). There were no significant relationships between all otolith elemental ratios and TL (*p* > 0.05), suggesting no evidence for the effect of growth on otolith elemental incorporation in the Japanese eel. No significant difference in otolith elemental ratios between male and female was found (*p* > 0.05). The classification accuracy by means of QDFA with jackknife cross-validation showed 50% for the three years groups using all six elemental ratios (Figure 2), suggesting less temporal variability of TEF in Japanese eel otoliths.

**Figure 3:**
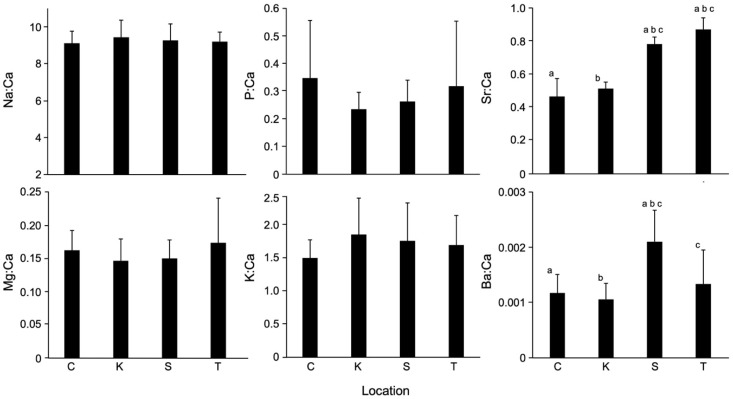
Otolith elemental ratios (mean ± SD) in the Japanese eel (*Anguilla japonica*) collected in four aquaculture farms in East Asia. C: China; K: Kagoshima Prefecture of Japan; S: Shizuoka Prefecture of Japan; T: Taiwan. The letters a, b and c indicate statistically significant difference at *p* < 0.001−0.0001.

In testing for spatial variability of TEF among four aquaculture farms, significant differences were found in Sr:Ca and Ba:Ca (*p* < 0.0001, df = 3, F = 11.725, 128.180), while no significant differences were found in Na:Ca, Mg: Ca, P:Ca, and K:Ca (*p* > 0.05, df = 3, F = 0.683–2.364) (Figure 3). In Sr:Ca, significant differences were found between China and Shizuoka (*p* < 0.0001), between China and Taiwan (*p* < 0.0001), between Kagoshima and Shizuoka (*p* < 0.0001), between Kagoshima and Taiwan (*p* < 0.0001), and between Shizuoka and Taiwan (*p* < 0.001) (Figure 3). There was no significant difference between China and Kagoshima (*p* > 0.05) (Figure 3). In Ba:Ca, significant differences were found between China and Shizuoka (*p* < 0.0001), between Kagoshima and Shizuoka (*p* < 0.001), and between Shizuoka and Taiwan (*p* < 0.001) (Figure 3). No significant differences were found between China and Kagoshima, between China and Taiwan, and between Kagoshima and Taiwan (*p* > 0.05). The classification accuracy using QDFA with jackknife cross-validation showed 80%.

**Figure 4:**
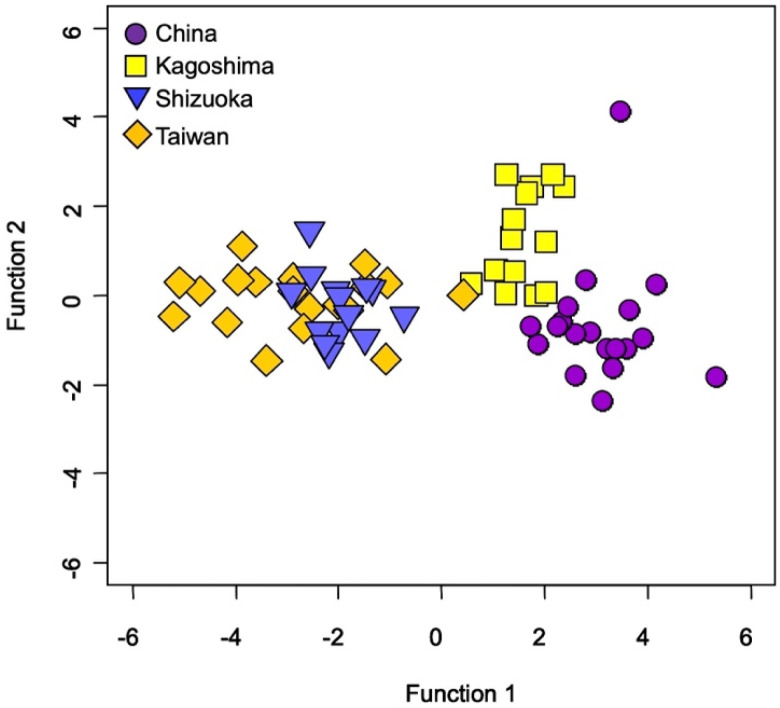
Quadratic discriminant function analysis among four aquaculture farms (China, Kagoshima Prefecture of Japan, Shizuoka Prefecture of Japan, and Taiwan) in East Asia based on all the otolith elemental ratios (Na:Ca, Mg:Ca, P:Ca, K:Ca, Sr:Ca, and Ba:Ca) in the Japanese eel (*Anguilla japonica*).

For the four aquaculture farm groups using all six elemental ratios (Figure 4), testing suggested high spatial variability of TEF in Japanese eel otoliths.

**Figure 5:**
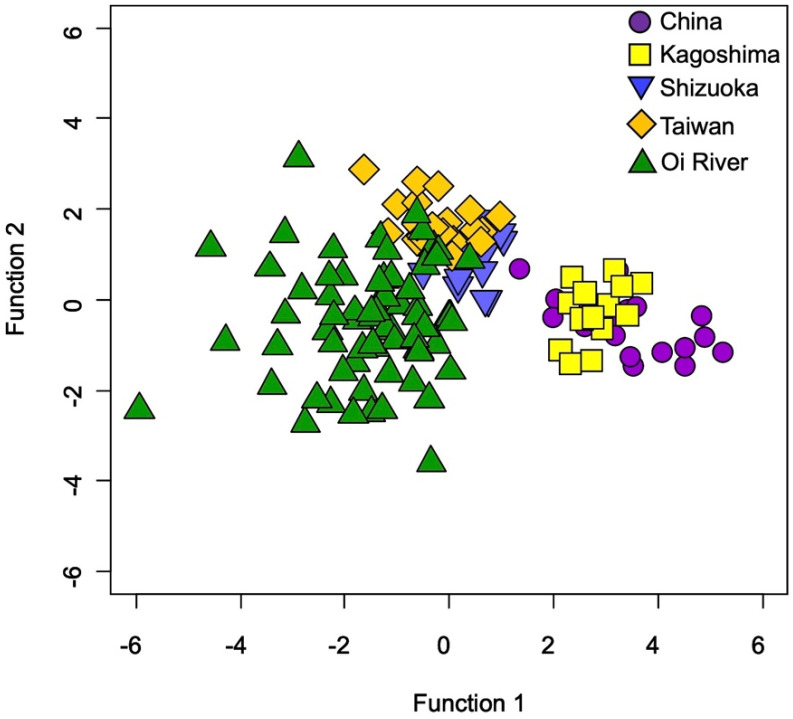
Quadratic discriminant function analysis among five locations in a wild habitat (Oi River) and four aquaculture farms (China, Kagoshima Prefecture of Japan, Shizuoka Prefecture of Japan, and Taiwan) based on all the otolith elemental ratios (Na:Ca, Mg:Ca, P:Ca, K:Ca, Sr:Ca, and Ba:Ca) in the Japanese eel (*Anguilla japonica*).

Testing for the QDFA with jackknife cross-validation using all data (one hundred thirty-seven specimens and six elemental ratios), including a wild habitat and four aquaculture farms, showed 82%, suggesting a high spatial variability of TEF in Japanese eel otoliths between wild and aquaculture environments (Figure 5).

No significant difference was found in TL over three years from a wild habitat (*p* > 0.05) and among four aquaculture farms (*p* > 0.05) (Table 1).

## 4. Discussion

Spatiotemporal variability of TEF in the Japanese eel otoliths in this study found less temporal variation but high spatial variation in East Asia. Otolith microchemistry is known to reflect various environmental factors such as salinity, temperature, water chemistry, diet, and interactive effects and physiological processes such as metabolism, growth, and reproductive stage. [35,36,37,38,39,40,41]. In the Japanese eel and other freshwater eels, otolith Sr is found to positively correlate with the Sr level in the ambient water, while the effects of temperature and diets on otolith Sr incorporation are minimal [42]. Arai and Hirata [43] suggested that otolith elemental compositions of Sr:Ca, Ba:Ca, Cr:Ca, and Mn:Ca reflect the water chemistry in the habitat of the Japanese eel. In the present study, wild Japanese eels were collected in the same area in a river for three years, and their biological characteristics were also similar throughout the three years. These Japanese eels would experience similar biotic and abiotic factors throughout their life history. Therefore, the elemental incorporation into otoliths would be spatially less variable over the three years. However, Japanese eels cultured in geographically different aquaculture farms would experience different ambient environmental factors rather than physiological processes. In cultured eels, their biological characteristics are almost the same because their marketable sizes are similar in East Asia. Eel culturing systems are different in each region and country, taking place either in a greenhouse or outdoor system [44]. The different culture systems would affect the incorporation of otolith elements, especially as an effect of water temperature. Otolith elemental composition in cultured eels would be more influenced by the regional water chemistry and temperature while the salinity effect would be minimal, as Japanese eels are cultured in freshwater ponds. Otolith TEF reflect environmental factors in each aquaculture and habitat environment. The present study shows that otolith TEF can be a reliable tool to discriminate the geographic origin in the Japanese eel.

In the present study, Sr:Ca and Ba:Ca were significantly different among locations (Figure 3). A considerable number of studies found that Sr and Ba concentrations were especially closely correlated to the concentrations of these elements in the ambient water environments [41,45,46]. In general, the otolith Sr:Ca is higher in seawater than in freshwater, while Ba:Ca is lower in seawater than in freshwater. A large number of studies have been applied to reconstruct the migratory history by means of otolith Sr:Ca signatures in diadromous fish, including freshwater eels. In the present study, however, all eels were collected in freshwater environments and hence, otolith Sr:Ca might be expected to be less variable among locations compared to Ba:Ca. Sr concentrations in freshwater environments would be influenced by regional geological features. Strontium is an alkaline earth element with Ba and is predominantly found in carbonate rocks composed of calcite, aragonite, and/or dolomite in sedimentary rocks [47]. Therefore, Sr concentrations in freshwater environments might be regionally variable. The present study suggests that Sr:Ca in the six elemental ratios examined can be a reliable indicator to discriminate the geographic origin of the Japanese eel and other freshwater fish.

Otolith TEF in the Japanese eel could successfully discriminate wild and cultured Japanese eels without external labeling and tagging. Interestingly, Oi River and an aquaculture farm are located a short distance from each other in Shizuoka Prefecture. The results suggest that otolith TEF in the Japanese eel would have high resolution to discriminate even in a geographically short range. The discrimination between wild and cultured Japanese eels is essential because cultured eels are used for restocking to recover the eel population. Therefore, to assess the usefulness of restocking and evaluate the population dynamics of the Japanese eel in the wild environment, accurate and reliable methods to discriminate wild and cultured Japanese eels are indispensable. Because the Japanese eel is a single panmictic population, molecular genetic markers are not available for discrimination. Otolith TEF in the Japanese eel would be a useful tool for the study of stock assessment in the future. In the present study, however, we did not examine chemical compositions of water samples, sediments, diet, feed, and other environmental factors such as temperature and salinity in each habitat and aquaculture farm. Therefore, further study is needed to examine the relationships between otolith TEF and biotic and abiotic factors to understand the mechanisms of elemental incorporation into otoliths in the Japanese eel. Nevertheless, the present study found that otolith TEF can be a reliable tool to discriminate the geographic origin of the Japanese eel. Otolith TEF would be a useful tool for traceability of the Japanese eel during global commercial trading.

## 5. Conclusions

This study examined the spatiotemporal variability of TEF in Japanese eel otoliths. The results showed that the otolith elemental composition in the Japanese eel showed lower temporal variability but high spatial variability in East Asia. Otolith TEF in the Japanese eel reflect the water chemistry in each aquaculture and habitat environment. The present study shows that the otolith TEF can be a reliable tool to discriminate the geographic origin of the Japanese eel. The Japanese eel is commonly cultured and consumed globally in the world as a delicacy [18,19,21]. The chemical composition of Japanese eel otoliths would be an essential tool for traceability during global eel-trading. Currently, the wild population of the Japanese eel is so depleted as to be on the brink of extinction [18,19,21]. Otolith TEF of the Japanese eel would also be a useful natural tag to assess the restocking effectiveness and the population dynamics at regional and global levels.

## Figures and Tables

**Table 1:** Total lengths (mm) of Japanese eel used in this study.

Location	Sample Size	Mean ± SD	Range
Wild habitat			
Oi River			
2007	30	508 ± 84.9	371–741
2008	20	572 ± 111	373–765
2009	20	516 ± 111	364–752
Aquaculture pond			
China	19	495 ± 19.1	466–551
Japan, Kagoshima	14	496 ± 22.7	462–545
Japan, Shizuoka	14	504 ± 20.2	458–528
Taiwan (Republic of China)	20	501 ± 20.9	463–539

## Data Availability

Not applicable.
